# Supplementary material for: Exploring Partners, Parenting and Pregnancy Thinking in Late Adolescents and Young Adults with Inherited Metabolic Disorders
Source: Pediatr Rep. 2025 May 13;17(3):56. doi: 10.3390/pediatric17030056 (PMC12101304; doi:10.3390/pediatric17030056)
Supplement: Supplementary file 1 [file pediatrrep-17-00056-s001.zip › pediatrrep-3554008-supplementary.pdf]

Table S1 – Patients' Genetic profile

	Type of IMD	Gene	Gene mutation
1	PKU	PAH	HMZ c.473G>A (p.Arg158Gln)
2	Argininsuccinic aciduria	ASL	HMZ c.1366C>G (p.Arg456Gly)
3	PKU	PAH	c.143T>C (p.Leu48Ser)/c.727C>T (p.Arg243Ter)
4	PKU	PAH	c.442-1G>A (IVS4+1 G>A)/c.842C>T (p.Pro281Leu)
5	MSUD	BCKDHB	HMZ c331C>T (p.Arg111X)
6	PKU	PAH	c.441+5G>T (IVS4 + 5 G>T) / c.842C>T (p.Pro281Leu)
7	PKU	PAH	N/A
8	GSD type 1A	G6PC	HMZ c.648G>T (p.Leu216=)
9	PKU	PAH	c.727C>T (p.Arg243Ter)/c.859C>G (p.Leu287Val)
10	Citrullinemia type 1	ASS	HMZ c.1168G>A (p.Gly390Arg)
11	OTCD	OTC	HEMZ c.119G>A (p.Arg40His)
12	LCFAOD (VLCAD)	ACADVL	c.950G>A (p.Gly317Glu)/ c.1708G>A (p.Val570Met)
13	PKU	PAH	c.442-1G>A (IVS4+1 G>A)/ c.1222C>T (p.Arg408Trp)
14	PKU	PAH	c.441+5G>T (IVS4 + 5 G>T)/ c.1066-11G>A (IVS 10-11 G>A)
15	PKU	PAH	c.442-1G>A (IVS4+1 G>A)/ c.1222C>T (p.Arg408Trp)
16	OTCD (carrier)	OTC	c.803T>C (p.Met268Thr)
17	LCFAOD (glutaric aciduria type 2, late onset)	ETFDH	c.1285+1G>A (p?)/c.1531G>A (Asp511Asm)
18	PKU	PAH	c.588_590 delTTC/IVS7+1G>A
19	PKU	PAH	c. 588_590 delTTC (p. Phe 39)/ c.727C>T (p.Arg243Ter)
20	PKU	PAH	c.898G>T (p.Ala300Ser)/IVS10-11 G>A
21	OTCD	OTC	HEMZ c.264A>T/p.Lys88Ans
22	Classic galactosemia	GALT	c.563A>G (p.Gln188Arg)/ c.-119_-116delGTCA
23	PKU	PAH	c.442-1G>A (IVS4+1 G>A)/c.842C>T (p.Pro281Leu)

HMZ: homozygosity; HEMZ, hemizygosity

PKU, phenylketonuria; UCD, urea cycle disorder; MSUD, maple syrup urine disease, OTCD, ornitin transcarbamilase deficiency; LCFAOD, long-chain fatty acid oxidation disorders; VLCAD, very long-chain AcylCoA dehydrogenase deficiency; GSD type 1A, glycogen storage disease, type 1A.
